# Improving Eating Habits at the Office: An Umbrella Review of Nutritional Interventions

**DOI:** 10.3390/nu15245072

**Published:** 2023-12-12

**Authors:** Aleksandra Hyży, Mariusz Jaworski, Ilona Cieślak, Joanna Gotlib-Małkowska, Mariusz Panczyk

**Affiliations:** Department of Education and Research in Health Sciences, Faculty of Health Science, Medical University of Warsaw, 00-581 Warsaw, Poland; aleksandra.hyzy@wum.edu.pl (A.H.); mariusz.jaworski@wum.edu.pl (M.J.); ilona.cieslak@wum.edu.pl (I.C.); joanna.gotlib@wum.edu.pl (J.G.-M.)

**Keywords:** workplace nutrition, behavioural interventions, cognitive interventions, employee well-being, organisational productivity

## Abstract

(1) Workplace nutrition interventions have garnered attention as a pivotal component of employee well-being and organisational productivity. However, the effectiveness of various intervention types remains inconclusive. This review aims to systematically evaluate the efficacy of cognitive, behavioural, and mixed nutrition interventions in the workplace, considering the nuances of intervention design, setting, and target demographics. (2) A comprehensive umbrella review was conducted, categorising the existing literature into person-oriented and environmental strategies. This review was prepared in line with the Joanna Briggs Institute methodology for umbrella reviews and the preferred reporting items for systematic reviews and meta-analyses reporting standard. (3) The analysis revealed a lack of definitive evidence supporting the universal effectiveness of any single intervention type. Nonetheless, behavioural and mixed interventions demonstrated more favourable outcomes as compared to purely cognitive strategies. Factors such as intervention design, workplace setting, and target group characteristics were identified as significant determinants of the intervention success. (4) The review emphasises the imperative for additional investigations that utilise evidence-based approaches to formulate sound guidelines for efficacious nutrition interventions in occupational settings. This review functions as a foundational framework for guiding both scholarly research and the pragmatic execution of nutrition programs in the workplace.

## 1. Introduction

The concept of well-being comprises health, happiness, and prosperity, including feeling mentally well, being satisfied with life, having a sense of purpose, and managing stress effectively [1]. Proper nutrition and a healthy diet are fundamental to good health and well-being. A balanced diet provides the necessary energy for daily activity as well as essential nutrients for growth and repair, promoting strength and health. It also facilitates the prevention of diet-related illnesses. An increasing body of research indicates that diet and nutrition have a substantial impact on mood and mental well-being, as well as on work performance [2]. This is of particular interest to employers, employees, and the public health sector, with well-being as an aspect of public health. The World Health Organisation collaborates with its member states and partners to promote the concept of well-being in global health and to achieve the 17 Sustainable Development Goals adopted by the United Nations [3].

Workplace well-being activities have a long history. Formal corporate well-being programs date back to the 1950s [4,5] and have observed rapid growth since the 1970s, mainly in the United States. Employers, wishing to reduce losses due to sickness absence, presenteeism (attendance at work despite illness) [6] or compensations, introduced preventive measures in the workplace. These measures resulted from the peculiarities of the US healthcare system, which does not entail public health insurance coverage; thus, healthcare costs are passed on to citizens and employers [7]. Initially, the implementation of well-being and health programs (e.g., as part of Employee Assistance Programs) aimed to prevent work-related illness and accidents. Over time, they also began to shape an employer-focused corporate culture oriented towards health promotion, enhancing the company’s brand and market position [8]. This became important in order to attract employees, whose expectations are constantly increasing; to create a company brand that is perceived as responsible and supportive of the employee; or to position the company through the awards given to top employers. This is also a result of the growing popularity of CSR (corporate social responsibility) and ESG (environment, social responsibility, and corporate governance). Over time, such an approach became recognised world-wide, and well-being initiatives have become a permanent part of the corporate culture of many companies [9,10,11].

Due to increased global health needs (staff shortages, challenges in obtaining healthcare services, aging population), for several years now, the World Health Organisation has identified the workplace setting as crucial for health promotion. The average employee spends one-third of the day in the workplace; thus, measures taken just in this environment seem reasonable and relatively easy to implement [12,13]. Currently, health-related benefits such as private health insurance, fitness perks, fruit and vegetable delivery to the office or lunch subsidies are among the most frequently offered, and their scope is steadily increasing. The reason for this is both the employers’ aspirations to distinguish themselves and the steadily rising employee expectations [9,11]. In addition, the workplace is also a space for socializing, sharing ideas and making friends and acquaintances, which can further contribute to the development of healthy eating habits [9,11].

When undertaking activities to promote health in the workplace, it is necessary to clearly define the target group, as well as the purpose and form of the activities (interventions) to be undertaken. Office workers are one of the most frequently addressed group of employee-directed health-related activities [10]. This is due to a number of factors that facilitate the design, implementation and evaluation of such interventions, e.g., a fixed pattern of work—work at similar times of the day and for a comparable amount of time, making most employees available at roughly the same place and time; or a similar range of duties—typically sedentary work that does not require the extra effort associated with, for example, having to stand for long periods of time or carrying objects. In addition, office workers in most cases are not shift workers, which has a huge impact on their circadian rhythm, meal times, eating habits and associated health risks. In addition, their privileged social position, higher education and higher earnings, and thus higher overall health competence, may be a major factor in the more frequent provision of well-being programs to this group of employees. All this makes it easier to establish a fairly homogeneous study group, allowing for the assessment of intervention effectiveness [10,11,14].

Another important step is to determine the purpose and form of the intervention. Overall, interventions can be divided into three main categories. The first category comprises cognitive interventions, which aim to increase nutritional knowledge and awareness of the impact of nutrition on health, e.g., through education, training or lectures. The second category comprises behavioural interventions, which are skill-giving interventions, i.e., interventions that focus on the recipient (e.g., through workshops or prevention programs) or that implement the changes needed to alter eating behaviour, as well as environmental interventions that focus on changes in access to or labelling of foods, e.g., providing fruit in the office or the colour-coding of cafeteria meals based on their nutritional value. The third category comprises mixed interventions, which combine cognitive and behavioural interventions. The cognitive interventions group may include, e.g., lectures (onsite and online) and e-learning courses. Behavioural interventions include, e.g., changing the availability of certain foods (limiting sweets in vending machines or providing fresh fruit and vegetables to the office), labelling healthy meals in the employee cafeteria with colours or symbols [15,16] or financial incentives for choosing healthy products [17]. Mixed interventions involve both components and may consist of workshops with a health care professional (doctor, nurse, nutritionist, public health specialist health educator, etc.), well-being programs combining lectures, workshops, exercise and dietary change, or, for example, diabetes prevention programs targeting the prevention and treatment of a particular disease [18,19,20].

The evaluation of the effectiveness of workplace nutrition interventions is a crucial component of their implementation [21]. Effectiveness can be measured using various indicators and methods. Cognitive interventions often involve pre- and post-intervention knowledge tests. The effectiveness of behavioural and mixed interventions can be assessed using work environment and economic indicators (e.g., absenteeism, presenteeism, costs) or health-related indicators (e.g., BMI, glucose levels, cholesterol levels, disease exacerbation, consumption of specific food groups). These indicators may be combined to provide a more comprehensive evaluation of the impact of interventions.

Currently, there is a notable lack of consensus on well-defined guidelines or recommendations for the design and implementation of workplace nutrition interventions. The proliferation of systematic reviews and meta-analyses on this subject presents a challenge in terms of consolidating the evidence and arriving at definitive conclusions. An umbrella review was determined to be the most appropriate approach to effectively collect and analyze existing data on nutritional interventions among office workers [22]. This method provides an overview of the evidence presented in available systematic reviews and meta-analyses, allowing for their comparison and evaluation. The umbrella review is optimal for exploring existing data, as it facilitates the discussion of various types of interventions, enabling conclusions to be drawn and gaps in the current state of knowledge to be identified.

### Aim and Research Question

The aim of this study was to synthesise the available scientific evidence regarding the effectiveness of various workplace-based nutrition interventions for office workers, as reported in secondary studies. An umbrella review was conducted to answer the following research questions:

- What kind of nutrition interventions are used in the office setting?
- What workplace nutrition interventions are effective for office workers?
- What are the factors contributing to the effectiveness of workplace nutrition interventions?

## 2. Materials and Methods

This review was prepared in line with the Joanna Briggs Institute (JBI) methodology for umbrella reviews [23] and the preferred reporting items for systematic reviews and meta-analyses (PRISMA) reporting standard [24].

### 2.1. Search Strategy

The overall search strategy was developed using the PICOS framework [25]:

- Population: office workers of all ages and genders;
- Interventions: dietary interventions, counselling, nutrition programs;
- Comparisons: not applicable;
- Outcomes: nutritional knowledge, economic indicators (e.g., absenteeism, presenteeism, costs) or health-related indicators (e.g., BMI, glucose levels, cholesterol levels, disease exacerbation, consumption of specific food groups);

In order to expand the scope of the search outcomes, no restrictions were imposed on the publication date. The literature search was carried out in November 2022, utilizing the PubMed/Medline, Embase, ProQuest, Scopus, and Web of Science Core Collection databases. Keywords were obtained through an initial search of articles on PubMed/Medline and the use of medical subject heading (MeSH) terms. The search strategies employed for each database are provided in Supplementary S1.

### 2.2. Eligibility Criteria

The inclusion criteria were as follows:

- Type of study: meta-analysis or systematic reviews that covered quantitative, qualitative, or mixed method studies that were peer reviewed.
- Type of participants: office workers.
- Type of controls: not applicable.
- Type of outcomes: nutritional knowledge, economic or health-related indicators.
- Language: papers written in English.

The following exclusion criteria were applied:

- Studies that included grey literature or professional guidelines.
- Studies that focused on groups of workers other than the target population.
- Studies that aimed to analyze the relationship between physical activity participation/adherence and the effects of nutrition interventions.
- Studies published in languages other than English.

### 2.3. Selection Process

All search results were imported into EndNote ver. 20 (Clarivate™, London, UK), i.e., reference management software. Duplicate entries were removed, and two researchers (AH, MP) independently screened the remaining articles based on their titles and abstracts using the Rayyan platform, an intelligent research collaboration tool for systematic literature reviews [26]. Discrepancies were resolved upon discussion, and if a consensus could not be reached, a third researcher was consulted. The full texts of the selected studies were then evaluated for inclusion in the study.

### 2.4. Data Collection Process

Data from the included reviews were extracted by both reviewers using a standardised data extraction sheet (Supplementary S2). The researchers divided the reviews for data extraction and subsequently checked 90% of each other’s extractions for accuracy. In cases of disagreement, a third reviewer was consulted. Information such as the title, authors, journal, publication year, review type (meta-analysis or systematic review), type of study (randomised controlled trials, quasi-experimental or observational studies), type of intervention(s) in the review (cognitive, behavioural or mixed), number of reviews included, total number of participants of primary studies included in the review, and data sources were extracted by one researcher and verified by the other.

### 2.5. Review Risk of Bias Assessment

The methodological quality of systematic reviews or meta-analysis of interventional studies was evaluated with the Assessing the Methodological Quality of Systematic Reviews 2 (AMSTAR 2) measurement tool [27]. Two reviewers independently rated the risk of bias and discrepancies were discussed with the third reviewer, if needed. The AMSTAR 2 measurement tool comprises seven critical domains, including: prospective registration of the protocol, search strategy, justification for the exclusion of particular studies, quality assessment of included studies, appropriateness of the analysis method, consideration of quality when interpreting results, and the presence of publication bias. In addition, there are nine non-critical domains. Each item is scored yes/partially yes/no/, and the overall methodological quality is classified as high (only ≤1 item in a non-critical domain rated as “yes”), moderate (>1 item in a non-critical domain rated as “yes”), low (1 item in a critical domain rated as “yes” regardless of the ratings in the non-critical domains), or critically low (>1 item in a critical domain rated as “yes” regardless of ratings in non-critical domains) (Supplementary S3).

### 2.6. Effect Measures

Given the broad scope of the review questions and the anticipated diversity, specific effect measures were intentionally left unspecified. The diverse nature of interventions, varying in scope and methodology across studies, necessitated an approach where specific effect measures were not pre-defined. This choice aligns with the practices in umbrella reviews where heterogeneity in study designs is a common challenge. Despite the unspecified effect measures, the methodological rigor of our approach ensures quality. We employed stringent selection criteria and comprehensive data analysis techniques to provide a robust synthesis of the available literature, preserving the integrity and applicability of our findings.

### 2.7. Synthesis Methods

Data were synthesised using a Microsoft Excel matrix. Information on the nutrition interventions in each of the included studies was extracted and categorisoned into three groups: cognitive, behavioural and mixed interventions. Within each of the defined groups, data were drawn on results, outcomes and final conclusions. Based on content analysis, the syntheses were summarised in a narrative format for each type of intervention [28].

## 3. Results

### 3.1. Search Process

**Figure 1:**
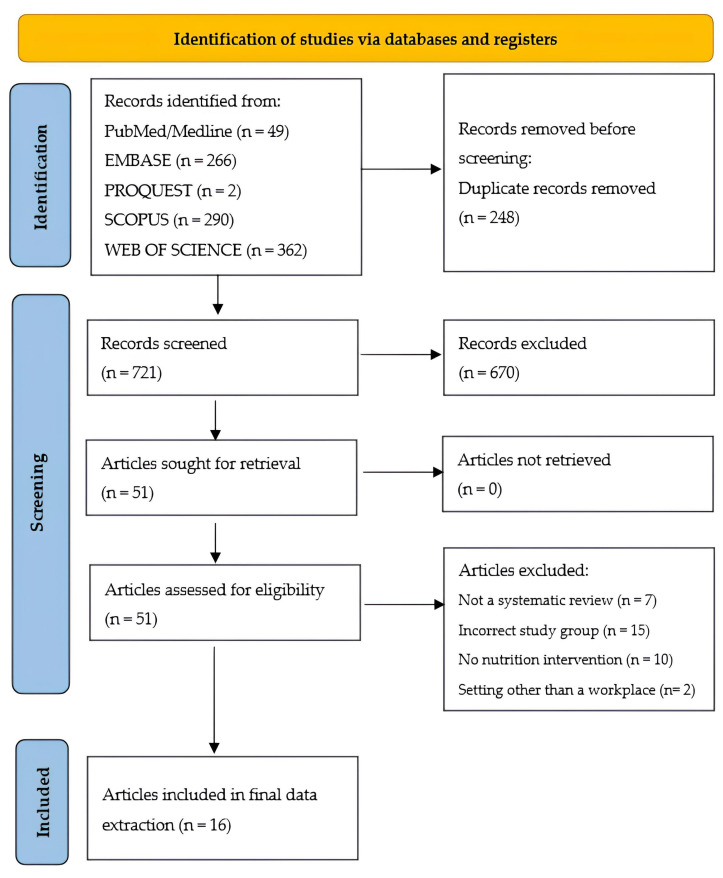
PRISMA flowchart of the systematic search and the selection process.

Based on specific keywords, 969 articles were identified from PubMed (Medline), Web of Science Core Collection, ProQuest, Scopus and Embase databases. After removing duplicates, 721 articles were screened for titles and abstract content. Subsequently, following the exclusion of 670 articles that did not meet the criteria, 51 were further subjected to full-text analysis. Ultimately, 16 systematic reviews (including four with meta-analysis) qualified for review. The remaining 35 reviews were rejected because the study group was different from that assumed in the inclusion criteria, there was no dietary intervention in the study, the review was not a systematic one (scoping or narrative review/overview), the intervention was in a setting other than the workplace, or it was a conference abstract (Figure 1).

### 3.2. Description of Included Systematic Reviews

The umbrella review comprised sixteen systematic reviews published from 2009 to 2022. These secondary studies included a total of 205 primary studies (after duplicate removal). The primary studies included in the reviews were published from 1976 to 2020. The results of the primary studies were mostly from the United States (116), Japan (12), Denmark (9), Germany (6), Australia (8), UK (8) and the Netherlands (11). Other studies were conducted in India, Tunisia, Singapore, Brazil, Sweden and Finland. There was only one primary international multicentre study.

The total number of primary study participants in those 16 systematic reviews exceeded 261,000, with the 5 largest studies involving more than 10,000 participants (57%). In some studies, data for analysis were obtained, e.g., from vending machines or cafeterias, hence the number of study participants was not determined. The characteristics of the 16 secondary studies included in this analysis are detailed in Table 1.

### 3.3. Reviews on Cognitive Interventions

It is important to note that, although none of the systematic reviews included in the analysis focused exclusively on the systematic evaluation of cognitive interventions, these reviews include seven primary studies with this type of intervention. The most common interventions were one-on-one counselling (in person or via electronic means of communication such as email or telephone) or lectures on nutrition education. Two studies involved enhanced nutritional education [39,40], of which one placed special emphasis on cultural differences (a study involving African-American women). The results of these studies are inconclusive—some emphasise the need to supplement cognitive intervention with behaviour change components [41,42], while others show the effectiveness of lectures or education alone, especially if personalised [43,44]. Secondary studies describing mixed interventions have shown that cognitive interventions alone, while more frequent, easier and cheaper to implement, are not effective unless supplemented with elements of support and behaviour change [18].

### 3.4. Behavioural Intervention Reviews

Behavioural interventions were discussed in five systematic reviews. The most common were: facilitating access to healthy foods (most commonly fruit and vegetables) or modifying diets [15,16,37], e.g., making fruit and healthy snacks (nuts, whole-grain cereals, vegetable and fruit snacks) available in the workplace; modifying the composition of meals (less fat, more vegetables in meals) available in the employee canteen or restaurant; modifying the size of the portions available; financial benefits or discounts based on the choice of specific products; labelling products with, e.g., colours, based on to the composition of a particular meal [17]; or multi-component programs aimed at changing eating habits in patients with diabetes or prediabetes [30]. Although the available studies demonstrate low strength of evidence, interventions aimed at changing the intake of some of the products available in vending machines or employee cafeterias (POP—point of purchase) or those increasing the availability of fresh fruit and vegetables appear as the most effective in terms of modifying employee diets. A detailed description of systematic reviews regarding behavioural interventions is presented in Table 2. To provide more specificity, this review includes an overview of common effect measures observed in the studies, such as changes in nutritional knowledge, dietary patterns, BMI, and productivity-related outcomes, offering insights into the varied nature of intervention impacts.

The most common endpoints evaluating the effectiveness of the intervention were: change in body weight or composition, consumption of specific products, or the results of biochemical tests (e.g., glucose, total cholesterol, LDL, HDL). The findings have been inconclusive and do not allow us to make clear recommendations for workplace interventions. The situation is particularly challenging due to the variety of factors such as age, gender, place of residence, type of work, and cultural aspects. Moreover, the significance of longer follow-up periods is emphasised, as it is not always possible to observe the effects of interventions, not because of their absence, but because of their emergence towards the end of the follow-up period. Depending on the study, there have been one-time interventions (with no follow-up period) and interventions with a short follow-up period (up to three months), for to up to two years of the follow-up. To address this, it is necessary to conduct further well-designed, methodologically sound studies capable of producing high quality results. It is also important to educate employers about the importance of such studies, for the sake of future research and interventions with measurable effectiveness.

### 3.5. Reviews on Mixed Interventions

The umbrella review comprised 11 systematic reviews of studies with mixed interventions, both cognitive and behavioural. The most common endpoints evaluating the effectiveness of the interventions were, as with behavioural interventions, the change in body weight or composition, consumption of specific foods, or the results of biochemical tests (e.g., glucose, total cholesterol, LDL, HDL). The interventions encompassed comprehensive programs including lectures or workshops, individual consultations with a specialist, coaching (cognitive interventions) and environmental interventions (changing the menu in the employee cafeteria, labelling products, changing the composition of products available in vending machines and access to fresh fruit and vegetables in the workplace), exercise programs, financial benefits (discounts on healthier products or a certain amount of money for reaching a goal set in the study), self-management interventions and other behavioural interventions. The interventions described applied to both the general population of office workers (7 reviews) and specific groups of employees: women [35], overweight and obese individuals [20,38], individuals at risk for type II diabetes [31] and individuals at risk for the metabolic syndrome [18]. A detailed description of reviews focused on mixed interventions is presented in Table 3.

Studies and interventions involving only women, emphasised the role of social factors, the methodology of intervention, and intervention tailoring to the needs of the specific group, all of which have an influence on intervention effectiveness [35]. For employees at risk of type II diabetes, the health benefits of implementing Diabetes Prevention Programs could be observed, but since the quality of evidence is low, emphasis is put on the need for further research and education in this area [30,31]. Research on overweight and obese employee populations points to the need for further, better quality studies [38] and the need to adapt the intervention delivery method to changing living conditions; advancing technologies, such as social media and virtual assistants Lee et al. (2022) [20], among office workers at risk for the metabolic syndrome, mixed or behavioural interventions appear to be more effective than cognitive interventions [18]. Other studies show little effect or low strength of evidence for the effectiveness of interventions. The authors highlight the small number of available studies or errors in their design and conduct (e.g., lack of consideration of the needs of a specific group, insufficient follow-up period). As with behavioural interventions, the difficulty in evaluating the effectiveness of interventions lies in the multiplicity of factors such as age, gender, place of residence, type of work, cultural factors or comorbidity (which run differently in everyone) conditioning inclusion in the intervention. Interventions in employee canteens or dietary changes combined with counselling and nutrition education appear to be the most effective, although what is highlighted is the need for further research to confirm the evidence as well as the introduction of longer follow-up periods, as those assumed in particular studies may not have been sufficient to observe the effects of the interventions, which does not necessarily mean that they did not occur at all. There is also emphasis on the importance of personalised interventions (e.g., in terms of the topic or selection of the intervention provider) and on the development of new technologies and the opportunities they offer for nutrition intervention and education.

## 4. Discussion

This is the first umbrella review which discusses the effectiveness of workplace nutrition interventions. There are more and more employee well-being initiatives introduced by employers. They embrace a wide range of health- and wellness-related aspects, e.g., physical activity, mental health, substance abuse prevention, and guidelines for proper nutrition. The issue is increasingly important and interventions are expected to be undertaken, especially for office workers (white-collar professionals). Nevertheless, public health specialists and practitioners implementing them face a substantial challenge to design them in such way so that they are effective, evidence-based and cost-effective/cost-efficient.

The narrative synthesis has demonstrated that behavioural and mixed (cognitive-behavioural) interventions are more effective rather than solely cognitive ones. Therefore, it seems reasonable to promote those interventions that involve comprehensive well-being programs, personalised consultations and environmental interventions, such as menu modifications or improving access to healthy snacks at the workplace. A properly designed intervention needs to account for the needs and characteristics of its future participants. When designing workplace nutrition interventions, it is also important to consider employee diversity. Factors such as employee sex, religion, economic or social status, among others, may influence the overall participation and effectiveness. The analysis of employee needs and abilities should also be taken into consideration when designing interventions so that they are prepared ‘with’ the employees instead of just ‘for’ them.

Apart from the employees, employers also should be engaged into designing interventions as well. After all, they are the ones who make the final decision and provide financial coverage for the intervention which may offer better access to healthy products or improve consumption patterns of the employees. This may further improve employee health and limit the costs of healthcare providers as well as build better workplace organisational culture. Then, either as a continuation or a separate intervention, it may be beneficial to reduce access to snacks such as sweets, crisps and sweetened beverages found in vending machines or canteens, and replace them with fruit and vegetables for the employees.

In recent years, an increase in overweight and obesity rates has been observed, especially after the COVID-19 pandemic [45]. This is particularly noticeable among office workers with sedentary jobs which may contribute to the development of diseases of affluence such as diabetes or the metabolic syndrome. Therefore, the implementation of disease prevention programs in the workplace may improve employee health. As it was observed, these programs featured comprehensive approaches to those diseases and combined the aspects of nutrition, physical activity and counselling [18,30,31]. They are considered to be slightly effective but with no major improvement. Nevertheless, the obtained results are still insufficient to properly assess the effectiveness, as flaws can be found both in the design of the interventions themselves as well as in data quality.

Though no systematic reviews discussing only cognitive interventions were included in the umbrella review, there are some primary studies available that describe the effectiveness of this type of intervention. As cognitive interventions mainly include education in the form of lectures or individual consultations, they are not considered effective if they do not involve behavioural change [41,42]. This is because dietary choices are determined by a number of factors, rather than just knowledge, and include environmental factors (e.g., availability of food, social and cultural practices, price and advertising of food), intrapersonal factors (e.g., beliefs, attitudes), interpersonal factors such as friends and family relations, experience with food, and biologically determined behavioural predispositions [46]. Therefore, it is difficult to imagine that solely offering knowledge will profoundly alter one’s dietary choices. Cognitive interventions are very often cost-effective, easier to organise and implement. Moreover, they do not require special tools, buying and transporting food to the workplace, and there are also fewer people involved in the execution of such an intervention. In most cases, it is enough to involve one employee and one speaker to run the lecture or consultations. Nonetheless, it has been suggested that for cognitive interventions to be more effective, they should be a part of comprehensive solutions and not performed on their own [18].

In order to properly evaluate the effectiveness of behavioural interventions, they should be categorised into person-oriented and environmental interventions. The first group comprises individual counselling, workshops, behavioural prevention programs or financial incentives. As for workshops and counselling, there is no sufficient data to fully confirm their effectiveness [17,30]. This is because the effects may vary from person to person, as each employee has different needs and meeting them all may be challenging. The intervention should be performed by a specialist who understands the basics of nutritional education and counselling not only in the field of nutrition, but also in the field of social psychology, health education, anthropology and economics [46]. Financial incentives such as lower prices for healthy food or discounts for healthy snacks are relatively new and less commonly used interventions, as they require considerable financial coverage by the employer. Therefore, there is limited information to assess their effectiveness [17]. Nonetheless, they may be promising in the following years, especially when, with more and more employees underscoring the financial aspect of shaping their nutritional habits and benefits at the workplace that would satisfy them, as well as the fact that prices of healthy food are much higher compared to unhealthy food, this possibly becomes a significant barrier for the employees to buy healthy food themselves [47,48].

Environmental interventions are employed when aiming to induce behavioural changes and focus on food accessibility, e.g., more access to healthy foods such as fruit and vegetables and less access to unhealthy snacks in canteens or vending machines; food labelling, e.g., using colours to highlight nutritional value and encourage healthier choices; or collecting something, e.g., like stamps or points, to document health-promoting behaviours. Offering a special diet in the workplace (e.g., ready-to-consume meals, changing the menu in the canteen) may be effective, but has its limits. After all, employees do not spend their entire day at work and their nutritional habits are shaped in other settings as well. Still, there is not enough evidence to fully confirm or deny the effectiveness of these interventions [16]. The same principle applies to providing fruit and vegetables in the workplace. Their consumption may increase on-site, but not elsewhere. Thus, ensuring access to healthy snacks may be even more important with the steadily growing costs of food [48]. There is more research needed in this area, with special attention placed on intervention design and employer education [15,30].

As for mixed interventions, many components bear resemblance to those in cognitive and behavioural interventions. Nonetheless, mixed interventions also focused on some issues that may bring new light to workplace interventions, i.e., the use of technology in self-control and self-regulation health interventions in the workplace as well as focus on specific groups of employees.

Technological advancements and possibility of their implementation, e.g., in the form of video-consultations, wearable devices (e.g., smart watches, smart phones, sensors) or nutrition apps, draw from the self-control intervention model which facilitates the change of dietary habits. Wearables and apps appear to be effective tools, as one can always have them close; however, there is no collective agreement or guidelines on this matter. Nonetheless, this could pose a challenge, especially to employees with little or no digital competence, such as the elderly employees near retirement or other employees vulnerable to digital exclusion. This field being relatively novel, there is little theoretical research addressing such issues, which makes it worth exploring, especially with the growing popularity of end-user apps and devices [20,49].

Another action that may form a part of complex interventions is addressing the specific needs of particular groups of employees. As it turns out, in female-only groups addressing their specific needs and offering social support are more important than the focus on the intervention itself. Furthermore, the interpersonal and teaching skills of the instructor are key factors determining the effectiveness of the intervention. What has also been highlighted in the review is that the most effective interventions were not those run by a healthcare professional (or alternatively by someone outside the healthcare system who was only supported by a healthcare professional) [35]. This may serve as a warning sign for healthcare professionals, as it became evident that the effective communication and delivery of the intervention are equally or more important than qualifications of the instructor. This is particularly concerning at the present time, with the rising number of influencers discussing health issues. Therefore, it seems vital to prepare healthcare professionals to conduct interventions and wisely use social media to promote health-related matters [50,51]. Healthcare professionals should be well-prepared to implement interventions and learn the necessary know-how that will enable them to function not only as experts, but also as successful educators.

All that being said, most factors contributing to the effectiveness of nutrition interventions can be categorised into three aspects: the setting, the design and the group. The setting is mainly the place where the intervention is about to take place, with all of the tools needed to implement it. Starting from the office, the office kitchen or open space, it is important to adapt the surroundings to the needs of the employees and plans of the instructor. The requirements for the intervention should be taken into account, e.g., a laptop and a projector for the lecture, labels for the meals in the canteen, or a complete kitchen and supplies for cooking. It appears that the most effective settings are the ones nearest to the employees, e.g., wearables and apps, the canteen and the office, where they spend most of their working hours [16,20,29].

Intervention design should consider the type of intervention (cognitive, behavioural or mixed), timeframe and anticipated budget. As previously stated, behavioural and mixed interventions are considered more effective than just cognitive ones [18]. The assumed timeframe should enable the employees to benefit from the intervention fully; that is, the interventions should not be planned in summer months when most employees are on vacation or during important, e.g., national events. Also, the form (onsite, online) should be adjusted to the type of work in a particular office. Longer prevention programs might be more effective due to their extended length and increased availability [18,31]. The budget should be tailored to the employer’s financial resources and their specific needs. When there is sufficient funding for such interventions, it is considerably easier to design complex programs with many activities. However, low-cost and effective interventions can be found, such as food-labelling or changing the menu available in the canteen [17].

Focus on the background and specific needs of the target group is another crucial factor which may strongly influence the effectiveness of workplace nutrition interventions. The nutritional education model accounts for factors determining the motivation for change and action, i.e., past behaviours, demographics and the cultural context, food preferences and prior experience with food, personality, moods and emotions, media exposure, and other individual differences. All of these factors should be carefully considered and used for the proper design of the intervention to ensure its maximum effectiveness [46,52].

### Limitations

The main limitation of this umbrella review is the lack of good quality and full evidence studies confirming or denying the effectiveness of workplace nutritional interventions. Most reviews scored “low” on the AMSTAR2 scale, which suggests the need for further research and better-quality evidence.

## 5. Conclusions

With the limitations in mind, it is important to note that the interventions discussed lack sufficient evidence from high-quality studies. Therefore, there is a need for further, comprehensive research to be conducted using evidence-based methods and tools, to enable the comparison of study results.

The insights from this review might help in further research needed to establish the guidelines for effective future interventions. This paper serves as a foundation for further scientific research, but it also offers guidance for practitioners responsible for implementing workplace programs and interventions.

The researchers should take into account both the type of intervention (behavioural or mixed, rather than just cognitive) and the factors (demographic, economic, social and health) that may influence intervention effectiveness in a given study group. This will result in interventions which are better suited for the needs of employees and, as such, will produce better outcomes. The main challenge is to seek innovative interventions grounded in strong evidence-based practices, as well as theoretical frameworks, including psychological and learning factors.

For practitioners, this will also enhance the quality of interventions and will help to achieve the optimal cost-effectiveness and cost-efficiency, making it easier to design customised intervention programs from which employees genuinely benefit. It should also be emphasised that when designing or implementing interventions, practitioners should make more use of research or studies accessible for reference, e.g., standardised tools to compare intervention effectiveness. Future research with standardised effect measures is recommended to facilitate direct comparisons and meta-analyses. For workplace interventions to become truly evidence-based, practitioners should integrate the previously mentioned employees’ needs and values, their own experience and expertise, as well as research evidence provided in this review, with all of these factors being of equal importance.

## Figures and Tables

**Table 1:** Secondary studies included in the umbrella review.

Author (Year)	Number of Primary Surveys	Number of Participants	Type of Primary Research	Type of Intervention	Type of Review	Quality Assessment of Primary Research (Yes/No; Name of Tool)	AMSTAR2 Evaluation
Allan et al. (2017) [15]	22	N/A *	RCTs, Quasi-experiments	Behavioural	Systematic review	Cochrane Risk of Bias Tool	Low
Anderson et al. (2009) [29]	47	76,941 **	RCTs, Quasi-experiments, Observational studies	Mixed	Systematic review and meta-analysis	Community Guide	Moderate
Brown et al. (2018) [30]	22	35,197	RCTs, Quasi-experiments, Observational studies	Behavioural	Systematic review	Cochrane criteria	Low
Cabrera et al. (2021) [18]	13	5423	RCTs, Quasi-experiments	Mixed	Systematic review and meta-analysis	Not specified	Low
Fitzpatrick-Lewis et al. (2022) [31]	5	1494	RCTs, Observational studies	Mixed	Systematic review and meta-analysis	Cochrane Risk of Bias 1 tool	Low
Geaney et al. (2013) [16]	6	N/A *	RCTs, Quasi-experiments	Behavioural	Systematic review	Cochrane Collaboration’s risk of bias tool	Low
Ghobadi et al. (2022) [32]	8	1797	RCTs	Mixed	Systematic review	Cochrane Collaboration’s risk of bias tool	Low
Groeneveld et al. (2010) [33]	31	16,013	RCTs, Quasi-experiments	Mixed	Systematic review	Delhi list based tool	Low
Gudzune et al. (2013) [34]	9	76,465 **	RCTs, Quasi-experiments	Mixed	Systematic review	Downs and Black methodological quality assessment checklist	Low
Hendren et al. (2017) [19]	18	37,744	RCTs	Mixed	Systematic review	Quality characteristics and bias criteria were adapted from two previously published systematic reviews	Low
Lee et al. (2022) [20]	11	13,233	RCTs	Mixed	Systematic review	The Joanna Briggs Institute Critical Appraisal Checklist for Randomised Controlled Trials	Low
Madden et al. (2020) [35]	20	3311	RCTs, Quasi-experiments	Mixed	Systematic review	Cochrane Risk of Bias, ROBINS-I (risk of bias in non-randomised studies of interventions)	Low
Ni Mhurchu et al. (2010) [36]	16	N/A *	RCTs, Quasi-experiments	Mixed	Systematic review	A checklist adapted from a previous review	Low
Park et al. (2019) [37]	7	2854	RCTs	Behavioural	Systematic review and meta-analysis	Cochrane’s Risk of Bias	Low
Sandercock et al. (2018) [38]	23	41,867	RCTs, Quasi-experiments, Observational studies	Mixed	Systematic review	Quality Criteria Checklist from the Academy of Nutrition and Dietetics (AND) Evidence Analysis Manual	Low
Sawada et al. (2019) [17]	3	3013	RCTs	Behavioural	Systematic review	GRADE (Grading of Recommendations, Assessment, Development and Evaluation)	Low

N/A—Not Applicable; RCTs—Randomised Controlled Trials; AMSTAR2—Assessment of Multiple Systematic Reviews (version 2). * In some studies, the number of participants is not given, as the study was conducted in cafeterias; the intervention involved a change in products offered in vending machines, making it significantly difficult or impossible to determine the number of participants; or the number of participants is not given **—WHO review.

**Table 2:** Secondary studies with behavioural interventions included in the umbrella review.

Author (Year)	Description of the Intervention	Results	Implications
Allan et al. (2016) [15]	Environmental intervention (environmental intervention) affecting eating habits	For behavioural endpoints, 13 of 22 studies showed a significant effect on primary endpoints. For physical endpoints, some studies showed no difference in BMI or body weight, while others confirmed it.	The current state of knowledge does not allow for clear recommendations for introducing environmental interventions to change eating habits in the workplace.
Brown et al. (2017) [30]	Workplace well-being programs to prevent or treat diabetes (nutrition—cooking workshops, individual dietary consultations, dietary changes; physical activity pedometers, workout plans; smoking cessation; usually in combination)	The study demonstrated a steady improvement in health in biological measures, self-reported behavioural adherence measures, and psychosocial variables. The authors presented data that showed improvement in most cases.	Workplace diabetes prevention programs can be useful in reducing disease occurrence and progression, but better design of interventions is needed. Employer education and further research in this area are crucial.
Geaney et al. (2013) [16]	Change in the composition of meals available at work, change in portion sizes (usually reduction), changes in access to healthy products for employees.	All of the included studies showed changes in fruits and vegetables intake, but none showed an effect size greater than a half-portion increase in fruit and vegetables consumption.	Modification of workers’ meals may increase fruit and vegetables intake, but the strength of evidence is low.
Park et al. (2019) [37]	A nutritional intervention that limits the intake of energy and certain nutrients (carbohydrates or fats) or a balanced diet that ensures a normal supply of all nutrients	Employees’ body weight decreased significantly: WMD of −4.37 kg (95% CI −6.54 to −2.20; Z = 3.95, *p* < 0.001), so did BMI: WMD of −1.26 (95% CI −1.98 to −0.55) kg/m^2^, but it was statistically significant (Z = 3.47, *p* = 0.001), blood cholesterol and blood pressure values also declined—but the problem is the duration of the study and the quality of the data.	It is challenging to definitely state the effectiveness of interventions, but it is a good start for further research.
Sawada et al. (2019) [17]	Discounts on healthy food products or for a smaller portion ordered in the employee cafeteria, colour-coding of dishes (yellow, green and red),	No significant changes in BMI, blood cholesterol levels or changes in diet	Link between the intervention and the outcomes cannot be established; poor quality of evidence; a need for further research in this area.

CI—confidence interval; *p*—probability value; WMD—weighted mean difference; BMI—body mass index; Z—z-score statistics.

**Table 3:** Secondary studies with cognitive and behavioural interventions included in the umbrella review.

Author (Year)	Description of the Intervention	Results	Implications
Anderson et al. (2009) [29]	Environmental, educational or behavioural interventions to achieve and/or maintain a healthy body weight	There is evidence of a modest reduction in body weight as a result of workplace health promotion programs aimed at improving nutrition, physical activity or both. Program effects are consistent, with a net loss of 2.8 pounds (95% CI −4.63, −0.96) among workers at 6–12-month follow-up, based on the meta-analysis of nine RCTs. In terms of BMI, a net loss of 0.47 BMI (95% CI −1.02, −0.2) at 6–12 months was observed in six RCTs.	There is strong evidence of a consistent, although small, effect (weight loss), in both men and women. The research quality is lacking and indicates the need and room for more research.
Cabrera et al. (2021) [18]	Basic nutrition education and general nutrition counselling, implementation of a specific diet, or dietary changes, motivational changes and/or coaching, physical activity and stress and/or sleep quality management. Most of the interventions studied were partially or fully delivered online using online platforms and/or social media	The effects of nutritional interventions: reduction in waist circumference (−4.9 cm, 95% CI −8.0 to −1.7), systolic blood pressure (−6.5 mmHg, 95% CI −10.7 to −2.3), diastolic blood pressure (−1.9 mmHg, 95% CI −3.6 to −0.2), triglycerides (SMD −0.46, 95% CI −0.88 to −0.04) fasting glucose (SMD −0.68, 95% CI −1.20 to −0.15).	Nutrition interventions in the workplace are beneficial for employees with the metabolic syndrome in terms of preventing the disease and also improving health parameters. Interventions that affect health-related behaviours and attitudes, as well as employee motivation, are the most effective—purely educational interventions are the most common but do not yield the anticipated outcomes.
Fitzpatrick-Lewis et al. (2022) [31]	A diabetes prevention program or a program with 3 components of diabetes prevention (nutrition educator/coach, focus on nutrition and increased physical activity)	Participants in diabetes prevention programs were 3.85 times more likely to lose weight ≥ 5% (4 RCTs; RR = 3.85; 95% CI, 1.58 to 9.38; *p* < 0.05) and had a 9.36-fold greater chance of weight loss ≥ 7% (2 RCTs; RR = 9.36; 95% CI, 2.31 to 37.97; *p* < 0.05), a significant reduction in BMI was observed (5 RCTs; MD = −0.86; 95% CI, −1.37 to −0.34; *p* < 0.05). Interventions based on diabetes prevention programs were 2.12 times more effective in increasing physical activity compared to the control group (RR = 2.12; 95% CI, 1.06 to 4.25; *p* < 0.05).	The quality of these data are low to average. Due to doubts about the quality of the data and its limited availability, further research is needed in this area.
Ghobadi et al. (2022) [32]	Nutrition interventions: educational, counseling and environmental	Improvements in lipid indices (HDL, LDL) were observed. Available data say that while dietary interventions are effective in improving the cholesterol profile, they do not affect other variables.	More high-quality primary research is needed to confirm these relationships.
Gudzune et al. (2013) [34]	Self-management, dietary, physical activity and/or environmental intervention	There were no statistically significant changes in body weight and BMI in either women or men. However, those in the group with a higher BMI at baseline who received the intervention lost weight, while those in the control group gained weight (a statistically significant relationship).	There is weak to moderate evidence that self-management, dietary, physical activity and/or environmental interventions prevent weight gain in workers.
Groeneveld et al. (2010) [33]	Lifestyle or health promotion intervention with emphasis on nutrition and physical activity	There is no evidence that interventions of this type have a positive effect on body weight, blood pressure values, lipid profile or glucose levels. In contrast, there is strong evidence of their effect on fat reduction.	The effectiveness of interventions depends on whether the patients included in the study were at CVD risk or not, with interventions working better for those at risk.
Hendren et al. (2017) [19]	Greater availability of fruit and vegetables, subsidies for healthy produce, changing menus/portion sizes, education at point of purchase, combination of education and community intervention	It showed an increase in fruit and vegetables intake which was statistically significant in 13 out of 14 studies (*p* < 0.05). Only one study showed a statistically significant decrease (*p* = 0.007). Three studies produced mixed results. One study showed a significant increase in vegetable intake (*p* = 0.002) but no change in fruit intake (*p* = 0.78). Another study showed a significant increase in fruit consumption (*p* = 0.001) but no change in salad sales (*p* = 0.139).	Environmental interventions conducted at the employee cafeteria/canteen can increase fruit and vegetable consumption, but the lack of consistency in the available literature limits the development of specific recommendations.
Lee et al. (2022) [20]	Weight loss interventions carried out using electronic devices such as computers, tablets, smartphones, apps and personal electronic assistants	Video consultations appear to be more effective than face-to-face appointments, while wearable devices (telemedicine devices, smartwatches and smart phones) and apps have proven to be the most effective.	As technology advances, the form of the message has to be updated. Also, these interventions lack a theoretical foundation—indicating the potential for future research.
Madden et al. (2020) [35]	Lifestyle programs to improve diet, physical activity and weight-related factors	In mixed activities (diet + physical activity), interventions that were not led by a health worker (possibly a healthcare worker and someone who is not—at the same time) were more effective. Emphasis was placed on how the interventions were delivered and on responding to the needs of female employees.	Proper social support and the right choice of interventions are key to the effectiveness of interventions with female employees.
Ni Mhurchu et al. (2010) [36]	A weight loss or healthy eating intervention in the workplace, lasting a minimum of 8 weeks	None of the studies showed measurable effects on presenteeism, productivity and/or health care costs. Overall, the effects of dietary interventions were positive, but the self-reported nature of dietary assessment poses a high risk of error.	Nutrition interventions in the workplace have a positive, though small, effect on employees’ eating habits.
Sandercock et al. (2018) [38]	Physical activity and nutrition education	The results of some studies have shown statistically significant changes in body composition (lower BMI, body fat percentage and waist circumference). Even though changes in body composition have been confirmed in other studies, the results are not statistically significant. Six interventions showed no change, and one showed an increase in BMI.	Interventions affect the body composition of the study participants, but the strength of evidence is low. More studies with better endpoint determination are needed—the authors suggest, e.g., BIA.

CI—confidence interval, *p*—probability value, RCTSs—randomized controlled trials, BMI—body mass index, SMD—standardized mean difference, RR—relative risk, MD—mean difference, HDL—high-density lipoprotein, LDL—low-density lipoprotein, CVD—cardiovascular disease, BIA—bio-electrical Impedance Analysis.

## Data Availability

The datasets generated for this study are available on request to the corresponding author.

## Supplementary Materials

The following supporting information can be downloaded at: https://www.mdpi.com/article/10.3390/nu15245072/s1, Supplementary S1: search strategies (MeSH); Supplementary S2: data extraction sheet; Supplementary S3: Detailed assessment of multiple systematic reviews.
