# Supplementary material for: Improving Eating Habits at the Office: An Umbrella Review of Nutritional Interventions
Source: Nutrients. 2023 Dec 12;15(24):5072. doi: 10.3390/nu15245072 (PMC10745686; doi:10.3390/nu15245072)
Supplement: Supplementary file 1 [file nutrients-15-05072-s001.zip › Supplementary S1 - search strategies (MeSH).pdf]

		Key word	Synonim 1	Synonim 2	Synonim 3	Synonim 4	syntax
P	population	"Workplace"[Mesh] AND office	„work place”	„office worker*”	„working environment”	employees	(((((Workplace[MeSH Terms]) AND (office[Title/Abstract])) OR („work place"[Title/Abstract])) OR („office worker*"[Title/Abstract])) OR („working environment"[Title/Abstract])) OR (employees[Title/Abstract])
I	interwention	"Counseling"[Mesh]	education	„individual intervention”	consultation		(((Counseling[MeSH Terms]) OR (education[Title/Abstract])) OR („individual intervention"[Title/Abstract])) OR (consultation[Title/Abstract])
		"Diet"[Mesh]	nutrition				(Diet[MeSH Terms]) OR (nutrition[Title/Abstract])
		"Health Behavior"[Mesh]	„dietary behavior”	„dietary habits”	„food habits”		((("Health Behavior"[MeSH Terms]) OR ("dietary behavior"[Title/Abstract])) OR („dietary habits"[Title/Abstract])) OR („food habits"[Title/Abstract])
		„dietary intervention”					„dietary intervention"[Title/Abstract]
		"Nutritional Status"[Mesh]	"Nutrition Assessment"[Mesh]				("Nutritional Status"[MeSH Terms]) OR ("Nutrition Assessment"[MeSH Terms])

		"Food"[Mesh]	"Food and Beverages"[Mesh]	"Diet, Food, and Nutrition"[Mesh]			((Food[MeSH Terms]) OR (Food and Beverages[MeSH Terms])) OR (Diet, Food, and Nutrition[MeSH Terms])
C	----------	----------	----------	----------	----------	----------	----------
O	outcome	"Program Evaluation"[Mesh]	effectiveness				("program evaluation"[MeSH Terms]) OR (effectiveness[Title/Abstract])
		"Body Weight"[Mesh]	"Weights and Measures"[Mesh]	"Weight Loss"[Mesh]	"Weight Perception"[Mesh]	"Body Weight Changes"[Mesh]	(((("Body Weight"[MeSH Terms]) OR ("Weights and Measures"[MeSH Terms])) OR ("Weight Loss"[MeSH Terms])) OR ("Weight Perception"[MeSH Terms])) OR ("Body Weight Changes"[MeSH Terms])
		"Obesity Management"[Mesh]	„weight management"				("Obesity Management"[MeSH Terms]) OR („weight management"[Title/Abstract])
		"Overweight"[Mesh]	"Obesity"[Mesh]	"Body Mass Index"[Mesh]			"overweight"[MeSH Terms] OR "obesity"[MeSH Terms] OR "body mass index"[MeSH Terms]
		"Blood Glucose"[Mesh]					"blood glucose"[MeSH Terms]
		"Hyperlipidemias"[Mesh]	"Cholesterol"[Mesh]	"Cholesterol, LDL"[Mesh]	"Cholesterol, HDL"[Mesh]	"Triglycerides"[Mesh]	((((hyperlipidemias[MeSH Terms]) OR (Cholesterol[MeSH Terms])) OR ("cholesterol, ldl"[MeSH Terms])) OR ("cholesterol, hdl"[MeSH Terms])) OR

							(triglycerides[MeSH Terms])
		"Blood Pressure"[Mesh]					"blood pressure"[MeSH Terms]
		"Health"[Mesh]	„well-being"	„employee health"			"health"[MeSH Terms] OR "well-being"[Title/Abstract] OR "employee health"[Title/Abstract]
S	study type	"Meta-Analysis" [Publication Type]					"Meta-Analysis"[Publication Type] OR "Systematic Review"[Publication Type]
		"Systematic Review" [Publication Type]					

PUBMED/MEDLINE

P	(((((Workplace[MeSH Terms]) AND (office[Title/Abstract])) OR („work place"[Title/Abstract])) OR („office worker*"[Title/Abstract])) OR („working environment"[Title/Abstract])) OR (employees[Title/Abstract])
I	((((((((Counseling[MeSH Terms]) OR (education[Title/Abstract])) OR („individual intervention"[Title/Abstract])) OR (consultation[Title/Abstract])) OR ((Diet[MeSH Terms]) OR (nutrition[Title/Abstract]))) OR (((("Health Behavior"[MeSH Terms]) OR ("dietary behavior"[Title/Abstract])) OR („dietary habits"[Title/Abstract])) OR („food habits"[Title/Abstract]))) OR („dietary intervention"[Title/Abstract])) OR (("Nutritional Status"[MeSH Terms]) OR ("Nutrition Assessment"[MeSH Terms]))) OR (((Food[MeSH Terms]) OR (Food and Beverages[MeSH Terms])) OR (Diet, Food, and Nutrition[MeSH Terms]))
C	-
O	(((((((("program evaluation"[MeSH Terms]) OR (effectiveness[Title/Abstract])) OR ((((("Body Weight"[MeSH Terms]) OR ("Weights and Measures"[MeSH Terms])) OR ("Weight Loss"[MeSH Terms])) OR ("Weight Perception"[MeSH Terms])) OR ("Body Weight Changes"[MeSH Terms])))) OR ("overweight"[MeSH Terms] OR "obesity"[MeSH Terms] OR "body mass index"[MeSH Terms])) OR ("blood glucose"[MeSH Terms])) OR (((((hyperlipidemias[MeSH Terms]) OR (Cholesterol[MeSH Terms])) OR ("cholesterol, ldl"[MeSH Terms])) OR ("cholesterol, hdl"[MeSH Terms])) OR (triglycerides[MeSH Terms]))) OR ("blood pressure"[MeSH Terms])) OR ("health"[MeSH Terms] OR "well-being"[Title/Abstract] OR "employee health"[Title/Abstract])
S	"Meta-Analysis"[Publication Type] OR "Systematic Review"[Publication Type]
PICOS	((((((((Workplace[MeSH Terms]) AND (office[Title/Abstract])) OR („work place"[Title/Abstract])) OR („office worker*"[Title/Abstract])) OR („working environment"[Title/Abstract])) OR (employees[Title/Abstract])) AND (((((((((Counseling[MeSH Terms]) OR (education[Title/Abstract])) OR („individual intervention"[Title/Abstract])) OR (consultation[Title/Abstract])) OR ((Diet[MeSH Terms]) OR (nutrition[Title/Abstract]))) OR (((("Health Behavior"[MeSH Terms]) OR ("dietary behavior"[Title/Abstract])) OR („dietary habits"[Title/Abstract])) OR („food habits"[Title/Abstract]))) OR („dietary intervention"[Title/Abstract])) OR (("Nutritional Status"[MeSH Terms]) OR ("Nutrition Assessment"[MeSH Terms]))) OR (((Food[MeSH Terms]) OR (Food and Beverages[MeSH Terms])) OR (Diet, Food, and Nutrition[MeSH Terms])))) AND ((((((((("program evaluation"[MeSH Terms]) OR (effectiveness[Title/Abstract])) OR ((((("Body Weight"[MeSH Terms]) OR ("Weights and Measures"[MeSH Terms])) OR ("Weight Loss"[MeSH Terms])) OR ("Weight Perception"[MeSH Terms])) OR ("Body Weight Changes"[MeSH Terms])))) OR ("overweight"[MeSH Terms] OR "obesity"[MeSH Terms] OR "body mass index"[MeSH Terms])) OR ("blood glucose"[MeSH Terms])) OR (((((hyperlipidemias[MeSH Terms]) OR (Cholesterol[MeSH Terms])) OR ("cholesterol, ldl"[MeSH Terms])) OR ("cholesterol, hdl"[MeSH Terms])) OR (triglycerides[MeSH Terms]))) OR ("blood pressure"[MeSH Terms])) OR ("health"[MeSH Terms] OR "well-being"[Title/Abstract] OR "employee health"[Title/Abstract]))) AND ((Meta-Analysis[Publication Type]) OR ("Systematic Review"[Publication Type]))

N = 49

EMBASE

P	'office worker':ti,ab,kw OR 'work place':ti,ab,kw OR 'workplace'/exp OR 'work environment':ti,ab,kw OR employee:ti,ab,kw
I	'counseling'/exp OR 'diet'/exp OR 'nutrition'/exp OR 'health behavior'/exp OR 'health behaviors'/exp OR 'nutritional status'/exp OR 'nutritional assessment'/exp OR 'food'/exp OR education:ti,ab,kw OR intervention:ti,ab,kw OR consultation:ti,ab,kw OR 'diet therapy':ti,ab,kw
C	-
O	'program evaluation'/exp OR 'body weight'/exp OR 'standard'/exp OR 'body weight loss'/exp OR 'weight loss program'/exp OR 'perception'/exp OR 'body weight change'/exp OR 'obesity management'/exp OR 'weight management'/exp OR 'obesity'/exp OR 'body mass'/exp OR 'glucose blood level'/exp OR 'blood glucose monitoring'/exp OR 'hyperlipidemia'/exp OR 'cholesterol'/exp OR 'low density lipoprotein cholesterol'/exp OR 'triacylglycerol'/exp OR 'blood pressure'/exp OR 'hypotension'/exp OR 'health'/exp OR 'wellbeing'/exp OR 'health insurance'/exp
S	'meta analysis'/exp OR 'systematic review'/exp
PICOS	P AND I AND O AND S

N = 266

PROQUEST

P	(Office AND MESH.EXACT("Workplace")) OR summary("work place") OR summary(„office worker*") OR summary(„working environment") OR summary(employees)
I	MESH.EXACT("Counseling") OR summary(education) OR summary(„individual intervention" OR „dietary behavior") OR summary(„dietary habits" OR consultation) OR summary(nutrition OR „food habits") OR MESH.EXACT("Diet, Food, and Nutrition") OR MESH.EXACT("Health Behavior") OR MESH.EXACT("Nutritional Status") OR MESH.EXACT("Nutrition Assessment") OR MESH.EXACT("Food and Beverages")
C	-
O	(MESH.EXACT("Program Evaluation") OR (MESH.EXACT("Body Weight Changes") OR MESH.EXACT("Body Weights and Measures") OR MESH.EXACT("Body Weight") OR MESH.EXACT("Body Weight Maintenance")) OR (MESH.EXACT("Body Weights and Measures") OR MESH.EXACT("Weights and Measures")) OR MESH.EXACT("Weight Loss") OR MESH.EXACT("Weight Perception") OR MESH.EXACT("Body Weight Changes") OR MESH.EXACT("Obesity Management") OR MESH.EXACT("Overweight") OR MESH.EXACT("Obesity") OR MESH.EXACT("Body Mass Index")) OR ((MESH.EXACT("Blood Glucose Self-Monitoring") OR MESH.EXACT("Blood Glucose")) OR MESH.EXACT("Hyperlipidemias") OR (MESH.EXACT("Cholesterol, HDL") OR MESH.EXACT("Lipoproteins") OR MESH.EXACT("Cholesterol") OR MESH.EXACT("Lipoproteins, HDL")) OR MESH.EXACT("Cholesterol, LDL") OR MESH.EXACT("Triglycerides") OR (MESH.EXACT("Blood Pressure") OR MESH.EXACT("Blood Pressure Monitors")) OR summary("well-being" OR "wellbeing") OR summary("employee health") OR summary(„weight management") OR summary(effectiveness))
S	MESH.EXACT("Meta-Analysis as Topic") OR MESH.EXACT("Systematic Reviews as Topic")
PICOS	P AND I AND O AND S

N = 2

SCOPUS

P	TITLE-ABS-KEY ("work place" OR "office worker*" OR "working environment" OR employees OR ("Workplace" AND office))
I	TITLE-ABS-KEY (counseling OR education OR "individual intervention" OR consultation OR diet OR nutrition OR "Health Behavior" OR "dietary behavior" OR "dietary habits" OR "food habits" OR "dietary intervention" OR "Nutritional Status" OR "Nutrition Assessment" OR food OR "Food and Beverages")
C	-
O	TITLE-ABS-KEY ("Program Evaluation" OR effectiveness OR "Body Weight" OR "Weights and Measures" OR "Weight Loss" OR "Weight Perception" OR "Body Weight Changes" OR "Obesity Management" OR "weight management" OR overweight OR obesity OR bmi OR "Body Mass Index" OR "Blood Glucose" OR hyperlipidemias OR cholesterol OR ldl OR hdl OR "Blood Pressure" OR triglycerides OR "Blood Pressure" OR health OR "well-being" OR wellbeing OR "employee health")
S	TITLE-ABS-KEY ("Meta-Analysis" OR metaanalysis OR "Systematic Review")
PICOS	((TITLE-ABS-KEY ("work place" OR "office worker*" OR "working environment" OR employees OR ("Workplace" AND office))) AND (TITLE-ABS-KEY (counseling OR education OR "individual intervention" OR consultation OR diet OR nutrition OR "Health Behavior" OR "dietary behavior" OR "dietary habits" OR "food habits" OR "dietary intervention" OR "Nutritional Status" OR "Nutrition Assessment" OR food OR "Food and Beverages")) AND (TITLE-ABS-KEY ("Program Evaluation" OR effectiveness OR "Body Weight" OR "Weights and Measures" OR "Weight Loss" OR "Weight Perception" OR "Body Weight Changes" OR "Obesity Management" OR "weight management" OR overweight OR obesity OR bmi OR "Body Mass Index" OR "Blood Glucose" OR hyperlipidemias OR cholesterol OR ldl OR hdl OR "Blood Pressure" OR triglycerides OR "Blood Pressure" OR health OR "well-being" OR wellbeing OR "employee health"))) AND (TITLE-ABS-KEY ("Meta-Analysis" OR metaanalysis OR "Systematic Review"))

N = 290

WEB OF SCIENCE

P	"work place" OR "office worker*" OR "working environment" OR employees OR ("Workplace" AND office) (Topic)
I	(counseling OR education OR "individual intervention" OR consultation OR diet OR nutrition OR "Health Behavior" OR "dietary behavior" OR "dietary habits" OR "food habits" OR "dietary intervention" OR "Nutritional Status" OR "Nutrition Assessment" OR food OR "Food and Beverages") (Topic)
C	-
O	("Program Evaluation" OR effectiveness OR "Body Weight" OR "Weights and Measures" OR "Weight Loss" OR "Weight Perception" OR "Body Weight Changes" OR "Obesity Management" OR "weight management" OR overweight OR obesity OR bmi OR "Body Mass Index" OR "Blood Glucose" OR hyperlipidemias OR cholesterol OR ldl OR hdl OR "Blood Pressure" OR triglycerides OR "Blood Pressure" OR health OR "well-being" OR wellbeing OR "employee health") (Topic)
S	("Meta-Analysis" OR metaanalysis OR "Systematic Review")
PICOS	"work place" OR "office worker*" OR "working environment" OR employees OR ("Workplace" AND office) (Topic) and (counseling OR education OR "individual intervention" OR consultation OR diet OR nutrition OR "Health Behavior" OR "dietary behavior" OR "dietary habits" OR "food habits" OR "dietary

	intervention" OR "Nutritional Status" OR "Nutrition Assessment" OR food OR "Food and Beverages") (Topic) and ("Program Evaluation" OR effectiveness OR "Body Weight" OR "Weights and Measures" OR "Weight Loss" OR "Weight Perception" OR "Body Weight Changes" OR "Obesity Management" OR "weight management" OR overweight OR obesity OR bmi OR "Body Mass Index" OR "Blood Glucose" OR hyperlipidemias OR cholesterol OR ldl OR hdl OR "Blood Pressure" OR triglycerides OR "Blood Pressure" OR health OR "well-being" OR wellbeing OR "employee health") (Topic) and ("Meta-Analysis" OR methanalysis OR "Systematic Review") (Topic)

N = 36
