# Supplementary material for: Improving Eating Habits at the Office: An Umbrella Review of Nutritional Interventions
Source: Nutrients. 2023 Dec 12;15(24):5072. doi: 10.3390/nu15245072 (PMC10745686; doi:10.3390/nu15245072)
Supplement: Supplementary file 1 [file nutrients-15-05072-s001.zip › Supplementary S2 - data extraction sheet.pdf]

First Author:

Corresponding author and email:

Publication title:

Journal:

Volume (issue):

Pages:

DOI:

----

1. Type of secondary study:

- Systematic review
- Systematic review and meta-analysis
- Other

2. Research question in the review according to PICOS:

P (population):
I (intervention):
C (comparison):
O (outcome):
S (study type):<br>• RCT<br>• pseudo-experiment<br>• observational (cohort or case-control study)<br>• cross-sectional study<br>• descriptive

3. How many studies were included in the review?:

4. How many participants there were in all primary studies included in the review (if listed)?

5. How was the quality of the primary studies included in the review assessed (name of the control list)?

6. What are the conclusions of the review (in meta-analysis effect size [OR, RR, MD] and confidence interval [95%CI] should be given)?

7. Decision on including the review into the umbrella review:

- include
- exclude (giving reason for exclusion)
