# Supplementary material for: Improving Eating Habits at the Office: An Umbrella Review of Nutritional Interventions
Source: Nutrients. 2023 Dec 12;15(24):5072. doi: 10.3390/nu15245072 (PMC10745686; doi:10.3390/nu15245072)
Supplement: Supplementary file 1 [file nutrients-15-05072-s001.zip › Supplementary S3.pdf]

**First author (year)**	**1**	**2**	**3**	**4**	**5**	**6**	**7**	**8**	**9**	**9a**	**10**	**11**	**12**	**13**	**14**	**15**	**16**	**Overall result**
Allan (2017)	y	**p y**	n	**p y**	y	y	**p y**	y	**y**	**p y**	n	**n m**	n m	**y**	n	**n m**	y	low
Anderson (2009)	y	**p y**	n	**p y**	y	y	**p y**	p y	**p y**	**p y**	n	**y**	y	**y**	y	**y**	y	moderate
Brown (2018)	y	**p y**	n	**p y**	y	y	**p y**	y	**p y**	**p y**	n	**n m**	n m	**y**	n	**n m**	y	low
Cabrera (2021)	y	**p y**	n	**p y**	y	y	**p y**	p y	**n**	**n**	n	**y**	y	**y**	y	**y**	y	low
Fitzpatrick-Lewis (2022)	y	**p y**	n	**p y**	y	y	**p y**	y	**p y**	**p y**	n	**n**	y	**y**	n	**n**	y	low
Geaney (2013)	n	**p y**	y	**p y**	y	y	**p y**	p y	**p y**	**p y**	n	**n m**	n m	**y**	n	**n m**	y	low
Ghobadi (2022)	y	**p y**	n	**p y**	y	y	**n**	y	**p y**	**o rct**	n	**n m**	n m	**y**	n	**n m**	y	low
Groeneveld (2010)	y	**p y**	y	**p y**	y	y	**p y**	p y	**p y**	**p y**	n	**n m**	n m	**y**	n	**n m**	n	low
Gudzune (2013)	n	**p y**	n	**p y**	y	y	**p y**	n	**p y**	**p y**	n	**n m**	n m	**y**	n	**n m**	y	low
Hendren (2017)	n	**p y**	n	**p y**	n	n	**n**	p y	**p y**	**p y**	n	**n m**	n m	**y**	n	**n m**	y	low
Lee (2022)	y	**p y**	n	**p y**	y	y	**p y**	p y	**p y**	**o rct**	n	**n m**	n m	**y**	n	**n m**	y	low
Madden (2020)	y	**p y**	n	**p y**	y	y	**y**	p y	**p y**	**p y**	n	**n m**	n m	**y**	y	**n m**	n	low
Ni Mhurchu (2010)	n	**p y**	n	**p y**	n	n	**n**	n	**p y**	**p y**	n	**n m**	n m	**y**	n	**n m**	y	low
Park (2019)	y	**p y**	n	**p y**	y	y	**p y**	p y	**p y**	**o rct**	n	**y**	y	**y**	y	**y**	y	low
Sandercock (2018)	n	**p y**	n	**p y**	y	y	**p y**	n	**p y**	**p y**	y	**n m**	n m	**y**	n	**n m**	y	low
Sawada (2019)	y	**p y**	n	**p y**	y	y	**p y**	p y	**p y**	**o rct**	n	**n m**	n m	**y**	n	**n m**	y	low

y – yes; n – no; p y – partial yes; n m – no meta-analysis conducted; o rct – includes only RCTs
bold – critical domain
